# Effect of Electrolysis Conditions on Electrodeposition of Cobalt–Tin Alloys, Their Structure, and Wettability by Liquids

**DOI:** 10.3390/molecules29133084

**Published:** 2024-06-28

**Authors:** Ewa Rudnik, Grzegorz Włoch, Monika Walkowicz

**Affiliations:** Faculty of Non-Ferrous Metals, AGH University of Krakow, Al. Mickiewicza 30, 30-059 Krakow, Poland

**Keywords:** anions, bath speciation, electrochemical methods, texture, surface wettability

## Abstract

The aim of this study was a systematic analysis of the influence of anions (chloride and sulfate) on the electrochemical behavior of the Co-Sn system during codeposition from gluconate baths. The pH-dependent multiple equilibria in cobalt–tin baths were calculated using stability constants. The codeposition of the metals was characterized thermodynamically considering the formation of various Co_x_Sn_y_ intermetallic phases. The alloys obtained at different potentials were characterized in terms of their elemental (EDS and anodic stripping) and phase compositions (XRD), the development of preferred orientation planes (texture coefficients), surface morphology (SEM), and wettability (water; diiodomethane; surface energy). The mass of the deposits and cathodic current efficiencies were strongly dependent on both the deposition potential and the bath composition. The morphology and composition of the alloys were mainly dependent on the deposition potential, while the effect of the anions was less emphasized. Two-phase alloys were produced at potentials −0.9 V (Ag/AgCl) and lower, and they consisted of a mixture of tetragonal tin and an uncommon tetragonal CoSn phase. The preferential orientation planes of tin grains were dependent on the cobalt incorporation into the deposits and anion type in the bath, while the latter did not affect the preferential orientation plane of the CoSn phase. The surface wettability of the alloys displayed hydrophobicity and oleophilicity originating from the hierarchical porous surface topography rather than the elemental or phase composition. The codeposition of the metals occurs within the progressive nucleation model, but at more electronegative potentials and in the presence of sulfate ions, a transition from progressive to instantaneous nucleation can be possible. This correlated well with the partial polarization curves of the alloy deposition and the texture of the tin phase.

## 1. Introduction

The codeposition of tin with cobalt was initiated in the first half of the 20th century [1]. The process was typically carried out using alkaline baths of the cyanide–stannate or pyrophosphate type, yielding white to dark grey deposits. The electrodeposition of cobalt–tin alloys has been further developed because of their good decorative [2,3,4,5,6,7] and anti-corrosive [8,9,10] properties to produce coatings with the appearance of a chrome finish, but for applications where high wear resistance is not required [11]. Such coatings were produced from acidic, neutral, and alkaline aqueous baths of various compositions (e.g., fluoride, sulfate, and sulfate–gluconate electrolytes) [1,2,3,4,5,6,7] or deep eutectic solvents [9,10]. Today, the importance of electrodeposited Co-Sn alloys has shifted to other areas. These include applications such as connector contacts [12] and solderable coatings [13], but electrodeposited Co-Sn alloys are primarily used as anode materials for lithium-ion batteries. The latter usage originates from the high specific reversible capacity, increased cyclability, and reduced pulverization of the alloys [14]. For this purpose, the Co-Sn deposits are prepared by various electrochemical techniques (galvanostatic, potentiostatic, pulse, brush plating, etc.) and using electrolytes of other types (i.e., aqueous chloride baths, with pyrophosphate, tartrate or citrate additives, or molten chlorides) to obtain thin-film anodes with various microstructures (macroporous, microcolumnar, nanocrystalline, etc.) [15,16,17,18,19]. Recently, electroplated Co-Sn-based multicomponent alloys have also been proposed as efficient electrocatalysts for water splitting and green hydrogen production [20,21]. The structural requirements of the deposits can be easily controlled by a selection of electrolyte composition and/or potential current conditions. The morphology and composition of the coatings govern further specific properties, thus opening up new potential areas of application for electrolytic Co-Sn alloys. 

Tin and its alloys can be electrodeposited from gluconate baths [2,3,4,22,23,24,25,26,27,28,29]. These are typically sulfate salt solutions [2,3,4,22,23,24,25,29] containing sodium gluconate as an inexpensive, non-toxic, and biodegradable additive for complexing Sn(II) ions (or other metal cations) over a wide pH range. Other gluconate baths with metal chlorides or a mixture of chloride and sulfate salts have rarely been studied, despite the possibility of changing the coating characteristics by simply substituting anions in the solution [26,27,28]. Therefore, the purpose of this study was to determine the role of anions in the electrodeposition of Co-Sn alloys from slightly acidic gluconate electrolytes. This research work is the first systematic comparative study on the influence of chloride and/or sulfate ions on the codeposition of tin with cobalt at different potentials, their effect on the composition, structure, and morphology of the alloys, and subsequently on the wettability of the coating surfaces by polar and nonpolar liquids. The latter feature is important for both the main applications of the Co-Sn alloys, i.e., as advanced electroplated coatings with a self-cleaning surface [30] and as an electrode material in lithium-ion batteries [31].

## 2. Results and Discussion

### 2.1. Bath Speciation

Cobalt and tin are metals with approvable differences in M/M(II) electrode potentials for codeposition as alloys from simple salt solutions. However, tin’s natural tendency to form long needle-like dendrites [25,32] requires the use of bath additives (inhibitors or complexing agents) to prevent local, very rapid growth of the metal and thus achieve more uniform coatings. Cobalt(II) and tin(II) ions in gluconate solutions are distributed as soluble species like free ions and neutral and/or charged complexes. The specific distribution of the individual forms depends mainly on the total concentrations of the metal ions, the type and concentration of other bath components, the stability of the complexes, pH, and temperature. The speciation of the electrolytes used in this study was examined by constructing equilibrium diagrams for bath component concentrations (see the Section 3) and the equilibrium constants [33,34,35,36,37] given in Table 1. 

The electrolytes (pH 3.5) contained metal chlorides or sulfates with sodium gluconate (as a complexing and buffering agent), ammonium salts, and boric acid (both as buffering components); thus, simple metal cations M^2+^ (more precisely, aqua complexes), as well as hydroxy M(OH)n^2−n^, chloride MCl_n_^2−n^, sulfate M(SO_4_)_n_^2−2n^, gluconate MGlu_n_^2−n^, and ammine Co(NH_3_)_n_^2+^ complexes, were taken into account in a pH range from 2 to 5. Tin(II) ions do not form complexes with ammonia in aqueous solutions, even in excess of the complexant [38]. Cobalt(II)-borane and tin(II)-borane species were not considered because their stability constants were not reported in the available literature.

**Figure 1:**
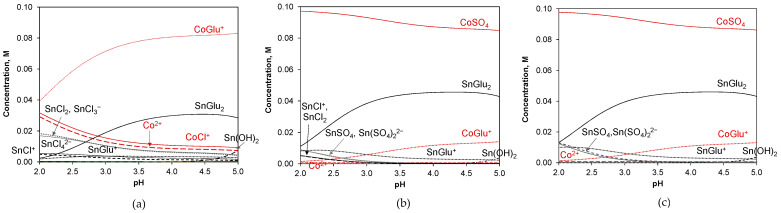
Equilibrium distribution of soluble metal species in gluconate solutions: chloride (**a**), chloride–sulfate (**b**), and sulfate (**c**) with compositions used in this study.

Figure 1 shows the equilibrium distributions of the metal species in the baths. It was found that at pH 3.5, the speciation of both sulfate-containing solutions is quite similar, with cobalt sulfate CoSO_4_ and tin gluconate SnGlu_2_ neutral complexes as the dominant species. Cationic complexes of the MGlu^+^ type existed in much smaller fractions. The chloride bath is more complicated in its ionic composition, with CoGlu^+^ and SnGlu^2+^ cationic complexes as the predominant species. A variety of tin chloride complexes and cationic cobalt forms (Co^2+^, CoCl^+^) can also occur. Despite the presence of ammonium salts in all electrolytes, the formation of cobalt-ammonia complexes can be disregarded for pH levels below 5, as they form only under more alkaline conditions [39,40,41]. Hydroxy complexes appear in very low concentrations (below 10^−5^ M), although tin(II) shows a tendency to hydrolyze, producing a small fraction of soluble Sn(OH)_2_ at pH values near 5.

Other ions involved in the complex species can also occur in their free forms, affecting the buffering properties of the solutions [42]. These include chloride ions Cl^−^ (0.7 M or 0.2 M in chloride or chloride–sulfate bath, respectively), sulfate ions SO_4_^2−^ (approx. 0.5 M in both sulfate-containing baths) and gluconate ions Glu^−^ (approx. 0.05 M in all baths). Ammonium cations NH_4_^+^ and boric acid molecules H_3_BO_3_ are assumed to exist in their total amounts at a pH of 3.5 due to their low dissociation constants [37,41]. 

The ionic composition of the electrolyte is useful for deducing the electrochemical reactions occurring on the cathode surface. However, it should be emphasized that the mechanism of electroreduction can involve other electroactive species as intermediates. These intermediates may exist in very low concentrations in the bulk electrolyte or be produced during subsequent steps of the reduction process.

### 2.2. Cyclic Voltammetry

**Figure 2:**
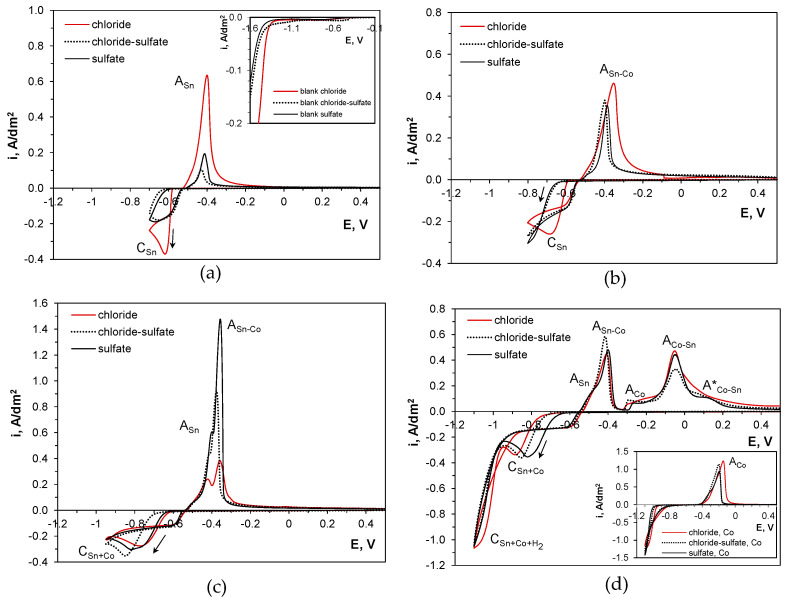
Cyclic voltammetry curves registered on glassy carbon electrode in gluconate baths for various vertex potentials: (**a**) −0.7 V (inset: blank solutions), (**b**) −0.8 V, (**c**) −0.95 V, (**d**) −1.1 V (inset: cobalt solutions). Arrow shows direction of forward scan, E vs. Ag/AgCl.

Figure 2 shows the gradual changes in cyclic voltammetric curves caused by different cathodic vertex potentials. An analysis of the curves indicates the potential ranges where tin and cobalt can codeposit.

Tin, being a more noble metal with high hydrogen overvoltage, starts to deposit at a potential of −0.6 V (Figure 2a). The cathodic reaction initiates faster in the chloride bath, while the presence of sulfate ions inhibits the process. These differences are further reflected by the varying heights of the anodic peaks A_Sn_, corresponding to the electrochemical dissolution of the metal phase. Although tin(II) predominantly exists as the gluconate complex SnGlu_2_ in the baths, it appears that the specific adsorption of free SO_4_^2−^ ions on the cathode surface disturbs the reduction of the metal ions, whereas chloride anions act as activating species [43]. Both inorganic anions can alter the structure of the double layer at the electrode surface and thus influence the charge transfer overvoltage. This effect is also noticeable in metal-free (blank) solutions (inset in Figure 2a). 

Shifting the vertex potential to more negative values of −0.8 V (Figure 2b) or −0.85 V enhances deposition from both sulfate-containing electrolytes but simultaneously hinders the process from the chloride one, as revealed by changes in the heights of the anodic peaks. This can be attributed to the simultaneous reduction of hydrogen ions, which is more likely in the presence of chloride than sulfate ions (inset in Figure 2a). During the backward scan, the anodic peak A_Sn-Co_ develops at a slightly more positive potential than A_Sn_, indicating the beginning of alloy codeposition during the forward scan. This is further evidenced by distinct double anodic peaks of tin dissolution A_Sn_ and the stripping of tin from the tin-rich alloy A_Sn-Co_ for the vertex potential of −0.95 V (Figure 2c). More negative vertex potentials enhance the incorporation of cobalt into the deposits, as reflected by a series of anodic peaks (Figure 2d). The peak A_Co_ at approx. −0.3 V can be attributed to cobalt oxidation, as it develops in a potential range where pure cobalt dissolves in a gluconate solution of similar pH (inset in Figure 2d) [42]. This is followed by the stripping of cobalt from the alloy phase A_Co-Sn_ at approx. −0.05 V and a smaller, wide decaying peak A*_Co-Sn_ at approx. +0.15 V, responsible for dealloying limited by solid-state diffusion of the reactant [44]. 

Interestingly, when the codeposition of the metals occurs, there are no significant differences in the anodic branches of the curves for different baths. This indicates that a mixture of tin, cobalt, and binary phases can be expected. These phases can be clearly distinguished due to the different electrochemical properties of each metal. Tin exhibits a high exchange current, resulting in only a slight difference between the cathode and anode peak potentials. Cobalt, on the other hand, has a low exchange current and high deposition overpotential, which means it undergoes deposition at much more negative potentials and then dissolves at much more positive potentials than tin. 

The simultaneous participation of different species (metal and hydrogen ions) in reactions on the electrode surface somewhat affects the course of the cathodic branches, indicating concurrent behavior. This is particularly important in the mechanism of cobalt codeposition, as it is accomplished via a hydroxide-type intermediate dependent on local pH changes on the cathode surface [45]. Moreover, a comparison of the electrochemical behavior of tin(II) and cobalt(II) species shows that both metals begin to deposit at more positive potentials (by about 0.1 V) than in single-metal gluconate baths with the same metal ion concentrations, i.e., −0.7 V for tin [26] and −1.0 V for cobalt [42]. This suggests that the kinetics of individual metal deposition changes when tin(II) and cobalt(II) species coexist in the electrolytes. This observation motivated a thermodynamic analysis of the Sn-Co codeposition and electrochemical experiments to clarify the performance of the systems.

### 2.3. Thermodynamic Analysis

During the simultaneous reduction of cobalt(II) and tin(II) ions,

Co^2+^ + 2e → Co (1)

Sn^2+^ + 2e → Sn (2)

an alloy is produced in the solid state:

*x*Co + *y*Sn → Co_x_Sn_y_ (3)

Consequently, changes in the quasi-rest potentials of the metals can be expected. If reaction (3) is at equilibrium, the change in free energy Δ*G* can be defined as follows [46]:

(4) $$∆G=-RTln\frac{a_{{Co}_{x}{Sn}_{y}}}{a_{Co}^{x}\cdot a_{Sn}^{y}}$$

where $a_{{Co}_{x}{Sn}_{y}}$, *a_Co_*, and *a_Sn_* are activities of Co_x_Sn_y_, cobalt, and tin in the deposit, respectively. The value of $a_{{Co}_{x}{Sn}_{y}}$ can be assumed as 1 for Co_x_Sn_y_ as a single phase in the deposit; hence,

(5) $$a_{Co}^{x}\cdot a_{Sn}^{y}=exp\left(\frac{∆G}{RT}\right)$$

The activities of both components in the deposit are not independent values, as an increase in the cobalt activity corresponds with a decrease in the activity of the second element. In the simplest case, when Co_x_Sn_y_ as the only compound in the system can coexist with pure metals (i.e., Co/Co_x_Sn_y_ and Sn/Co_x_Sn_y_), the following dependencies should be satisfied: $a_{Co}=1$, $a_{Sn}=exp\left(\frac{∆G}{yRT}\right)$ and $a_{Sn}=1$, $a_{Co}=exp\left(\frac{∆G}{xRT}\right)$. This results in shifting the quasi-rest potentials of the metal electrodes:

(6) $$E_{o,\;Co}=E_{Co}^{o}+\frac{RT}{2F}lna_{{Co}^{2+}}-\frac{∆G}{2xF}$$

and

(7) $$E_{o,\;Sn}=E_{Sn}^{o}+\frac{RT}{2F}lna_{{Sn}^{2+}}-\frac{∆G}{2yF}$$

where $E_{Co}^{o}$ and $E_{Sn}^{o}$ are the standard electrode potentials for cobalt and tin, respectively; $a_{{Co}^{2+}}$ and $a_{{Sn}^{2+}}$ are the activities of cobalt(II) and tin(II) ions at the electrolyte/deposit interface during electrodeposition, respectively.

Table 2 shows thermodynamic data for Co_x_Sn_y_ compounds [47] that can be found in the phase equilibrium diagram [48]. These include hexagonal CoSn, tetragonal CoSn_2_, and Co_3_Sn_2_ of two modifications: low-temperature orthorhombic α (stable up to approx. 500 °C) and high-temperature hexagonal β. Similar phases can be found in electroplated alloys [2,3,10,12,13]. The formation free energies Δ*G^o^* of the Co_x_Sn_y_ compounds are negative values, indicating that the quasi-rest potentials of both metals become more positive as a result of Co_x_Sn_y_ generation during electrodeposition. This trend is consistent with the CV data for the single (Co, Sn) and binary (Co-Sn) systems, although the theoretical shifts of the quasi-rest potentials are smaller than those found experimentally. However, this discrepancy is understandable since the quasi-rest potentials were compared with the results of dynamic measurements as a general case, independently of the bath composition and the phase composition of the alloys, which is unknown from the polarization data. Finally, it should also be noted that the actual conditions of alloy deposition depend on the kinetic properties of the components, as the polarization curve of the alloy does not necessarily represent the algebraic sum of the curves for the parent metals.

### 2.4. Potentiostatic Deposition

**Figure 3:**
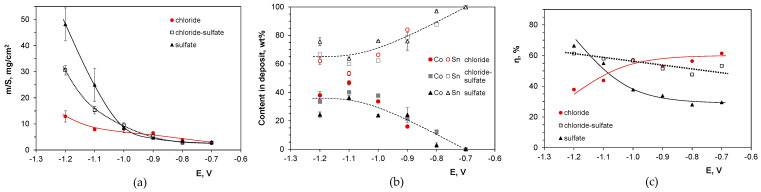
Influence of deposition potential (E vs. Ag/AgCl) on (**a**) mass of the deposit, (**b**) alloy composition, (**c**) cathodic current efficiency.

Figure 3 shows experimental data for the codeposition of the alloys. It was found that the mass of the deposits gradually increased with more negative potentials, with higher values for the baths containing sulfate anions, while electrodeposition in the chloride electrolyte was highly hindered (Figure 3a). These results are consistent with the current efficiency values (30–65%) (Figure 3c) and the potentiodynamic CV data (Figure 2) confirming the likely reduction of hydrogen ions in the presence of chloride rather than sulfate ions. The elemental composition of the deposits changed in a similar way with the potential, independently on the bath’s anions, stabilizing Co/Sn ratios in the deposits below −1.0 V at levels of about 35 wt% Co, i.e., close to a 1:1 Co/Sn atomic ratio. It seems that sulfate ions promoted the codeposition of tin, increasing its content in the alloys by a few percent (4–9 wt%) compared to the deposits produced from the chloride electrolyte. This may be attributed to the general bath speciation, which is more uniform and similar in both sulfate-containing systems. This can generate stable metal mass transport to the cathode, while the few positively charged cobalt species existing in the chloride bath (Figure 1a) can be moved faster towards the negative electrode, enhancing cobalt incorporation into the growing alloy layer. Moreover, faster hydrogen evolution in the chloride bath enhances the formation of CoOH^+^ species, which are crucial intermediates in cobalt electroreduction [45]. 

**Figure 4:**
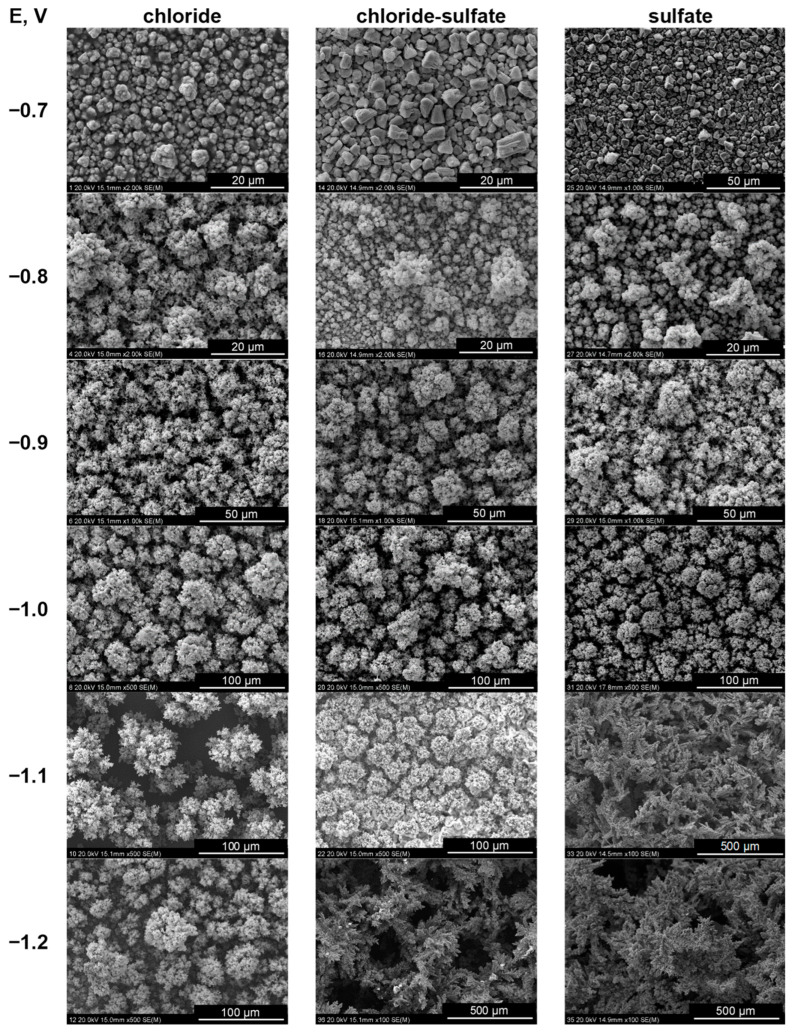
Surface morphology of Co-Sn deposits. E vs. Ag/AgCl.

**Figure 5:**
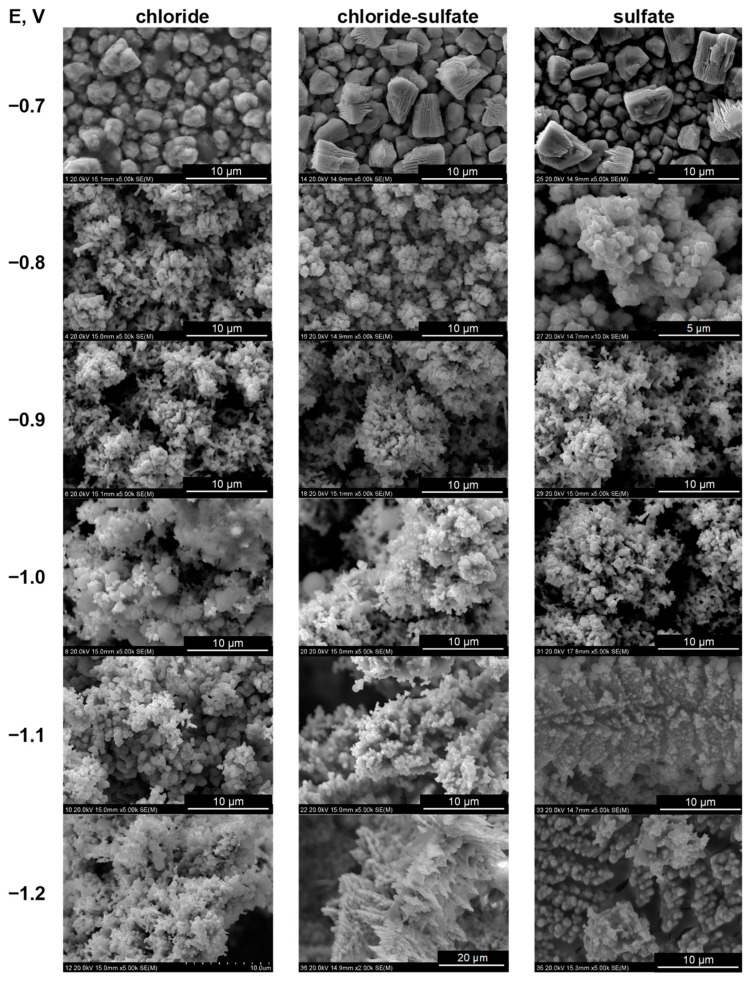
Morphology of Co-Sn deposits—structural details. E vs. Ag/AgCl.

Figure 4 shows gradual changes in the morphology of the deposits. At the most positive deposition potential of −0.7 V, compact tin deposits consisted of large, separate grains with shapes slightly dependent on the bath composition. Chloride ions favor more fine-grained deposits with a tendency to produce columnar structures. In contrast, in the presence of sulfate anions, polyhedral grains are formed. The codeposition of cobalt, even in small amounts, drastically changes the morphology of the alloys. The fine-grained deposits grow preferentially in a direction perpendicular to the substrate, resulting in porous columnar structures with a developed surface. In the presence of sulfate ions and at the most negative potentials, dendritic grains with shapes similar to fern leaves were grown. A detailed analysis of the surface structures (Figure 5) indicates the presence of a variety of grain shapes, such as globular (in chloride-containing baths), thin plates, and elongated formations, creating highly intricate columnar topographies. Their formation was also favored by the evolved hydrogen bubbles, as evidenced by deeper large oval pores.

**Figure 6:**
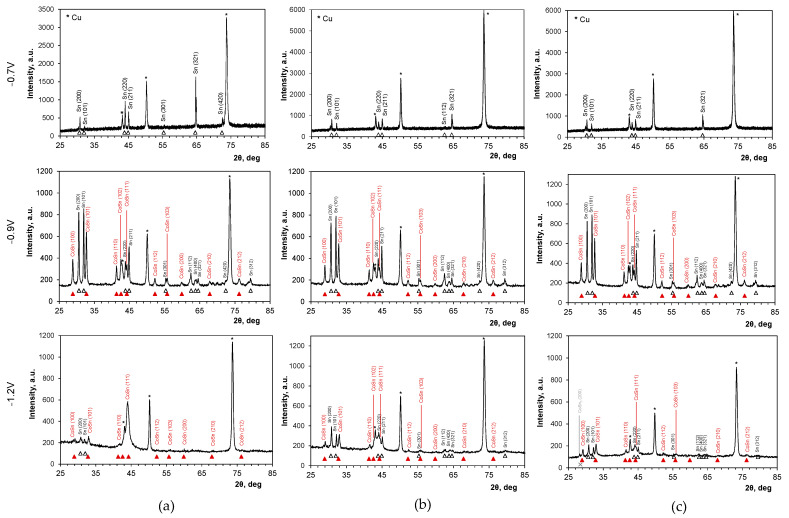
Exemplary XRD patterns for the Co-Sn deposits produced at different potentials from gluconate baths: (**a**) chloride, (**b**) chloride–sulfate, (**c**) sulfate (asterisk, triangle, and cross marks correspond to the standards used: copper JCPDS Card No. 00-004-0836, tin JCPDS Card No. 00-004-0673, and tetragonal CoSn phase [4] CoSn_3_ JCPDS Card No. 01-078-5055). E vs. Ag/AgCl.

The X-ray diffraction phase analysis of the alloys (Figure 6) identified a two-component mixture of tetragonal tin and a Co_x_Sn_y_ phase, which does not exist in the Sn-Co equilibrium diagram [48] and does not correspond to any available standards of the Co_x_Sn_y_ phases. This intermetallic is identical to the tetragonal CoSn phase first identified and described by Gomez et al. in 2001 [4] in alloys produced from a sulfate–gluconate electrolyte under certain conditions. The current study represents the first unequivocal confirmation of the formation of this uncommon phase, as similar evidence has not been reported in research papers published in the past two decades. At the most positive deposition potential (−0.7 V), where only cobalt traces were incorporated, a single β-tin phase was found. No peaks attributed to pure cobalt were detected. The existence of the CoSn_3_ phase in the deposits produced in the sulfate bath at the most negative potentials seems doubtful, as only one weak peak can be attributed to it.

Generally, the phase composition of Co-Sn electrodeposits depends on their chemical composition and typically can consist of cobalt, tin, and/or a series of Co_x_Sn_y_ phases (Co_3_Sn_2_, CoSn, CoSn_2_, CoSn_3_) [2,3,9,10,11,12,13,18,19,49,50,51], regardless of the electrolyte type (aqueous, ionic liquids, molten salts) and electrodeposition mode (galvanostatic, potentiostatic, pulse). However, only sporadic papers [13,17] have reported the presence of some XRD peaks attributed to unknown metastable phases in electrodeposited Co-Sn alloys with complex phase compositions.

A detailed analysis of the XRD data for the samples obtained in this study shows gradual changes in the structure of the layers induced by the electrodeposition potential. As the potential shifts to more negative values, crystalline deposits with sharp diffraction peaks transform into more fine-grained or partly amorphous structures, evidenced by decreased peak intensities and simultaneous broadening. This effect is particularly visible for the coatings produced from the chloride bath, where broad peaks at diffraction angles around 30° and 45° are observed for the potentials of −1.0 V and more negative. This observation aligns with findings from other studies [10,49], which noted that increased amounts of tin lead to increased crystallinity of deposits, whereas deposits richer in cobalt tend to become amorphous with broad peaks observed at 32° and 44°.

Modifying the plating conditions can indeed affect the crystal orientation of the deposits. Tetragonal β-tin layers with the highest peak intensity corresponding to the (321) plane were produced at a potential of −0.7 V. This texture is consistent with observations in electrodeposited tin layers [52,53]. The relative intensities of the tin diffraction peaks transformed with the deposition potential and increased cobalt content in the alloys. Simultaneously, the formation of the tetragonal CoSn phase with the same preferred orientation plane of (101) was identified for all baths. This finding aligns with the literature data [4] used as a reference. 

The preferential growth of the (*hkl*) planes can be quantitatively described using texture coefficients T_c_(*hkl*) [54]:

(8) $$T_{c}\left(hkl\right)=\frac{I(hkl)/I_{o}(hkl)}{\frac{1}{n}\;\sum \;[I(hkl)/I_{o}(hkl)]}$$

where *n* is the number of diffraction peaks measured; I(*hkl*) and I_o_(*hkl*) are the intensities of the particular (*hkl*) reflections of the analyzed sample and the reference standard, respectively. If the values of T_c_(*hkl*) are close to 1 for all the crystal planes considered, they indicate a randomly oriented crystallite structure similar to the reference standard in the investigated sample. Values of T_c_(*hkl*) ranging from 0 to 1 indicate the absence of grains oriented in that direction. Conversely, T_c_(*hkl*) values higher than 1 indicate the presence of many grains oriented in a specific direction, with a higher texture coefficient indicating more pronounced preferential growth of crystallites perpendicular to the considered (*hkl*) plane. 

Table 3 shows the texture coefficients for the electrodeposits obtained in this study, calculated separately for the main diffraction peaks detected in the samples. Specifically, four peaks were considered for tin, namely (200), (101), (220), and (321), and three for the CoSn phase, namely (100), (101), and (110). Given these considerations, the maximum possible values of T_c_(*hkl*) for the Sn and CoSn phases were 4.0 and 3.0, respectively. The analysis of the data confirms the presence of various preferential tin planes depending on cobalt incorporation. The (321) plane was preferentially developed only at −0.7 V in all baths, where traces of cobalt were detected in the deposits. The preferred orientation of the planes was influenced by two main factors, i.e., increased cobalt content in the deposits and the type of anion in the electrolyte. It appears that sulfate ions promoted the development of the (220) plane, whereas chloride ions induced the preferred orientation of the (200) and (101) planes. The preferred orientation of the (101) plane of the CoSn phase was consistently observed in all two-phase deposits, similar to that in the reference phase [4]. The existence of different preferentially oriented planes in the crystal lattice affects the growth of metal crystals of various shapes (Figure 4 and Figure 5) during electrodeposition [44]. This is usually attributed to the adsorption of various species (e.g., hydrogen atoms, metal intermediates, or ions from the bath) that selectively hinder the growth of certain planes and promote the formation of others [43,55]. Specifically, the selective concurrent adsorption of different cobaltous intermediates (CoCl^+^ and/or CoOH^+^) [45] appears to be responsible for the observed phenomena in this study, particularly when the cobalt content reaches at least a few or a dozen percent in the deposits.

### 2.5. Anodic Sweep Linear Voltammetry

**Figure 7:**
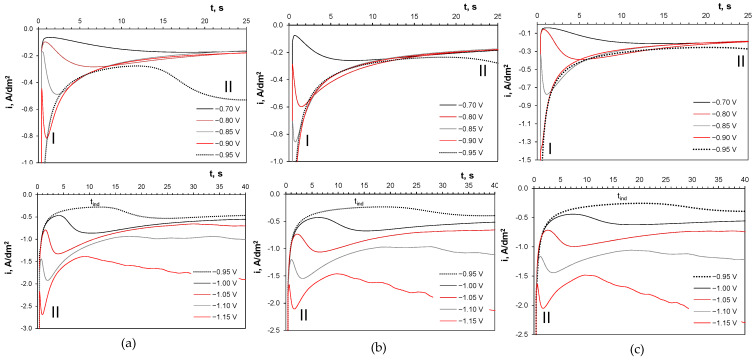
Chronoamperometric curves registered on GC in gluconate baths: (**a**) chloride, (**b**) chloride–sulfate, (**c**) sulfate. All labels (*t_ind_*, I, II) are explained in the text. E vs. Ag/AgCl.

Figure 7 shows chronoamperometric curves registered at constant cathode potentials for all baths, representing a 40-second accumulation step of the anodic sweep analysis. Two types of transients were observed. At potentials of −0.9 V and more positive, the curves showed a gradual increase in the cathodic current up to a maximum I, with their final part falling to a plateau. The shape of the *i-t* curves is typical for a three-dimensional nucleation and growth process occurring under mass transfer control [44]. For deposition potentials of −0.95 V and more negative, the chronoamperometric curves change. After charging the double layer, the cathode currents immediately fell to a plateau followed by the development of the nucleation peak II. This plateau corresponds to the induction time *t_ind_*, which shortens as the deposition potential becomes more negative. The induction period originates from the delayed nucleation of the solid phase and is longer in the presence of sulfate than chloride ions. Such an induction period is considered the time needed to reach a steady-state distribution of subcritical clusters associated with the adsorption–desorption of electroactive ions or the time needed for the appearance of active sites on the electrode surface [55]. During this stage, no significant nucleation can occur, and the small clusters may also dissolve back into the solution. The prolongation of this stage indicates some hindrance in the creation of active sites by adsorbed non-electroactive species (e.g., sulfate ions) or hydrogen. 

**Figure 8:**
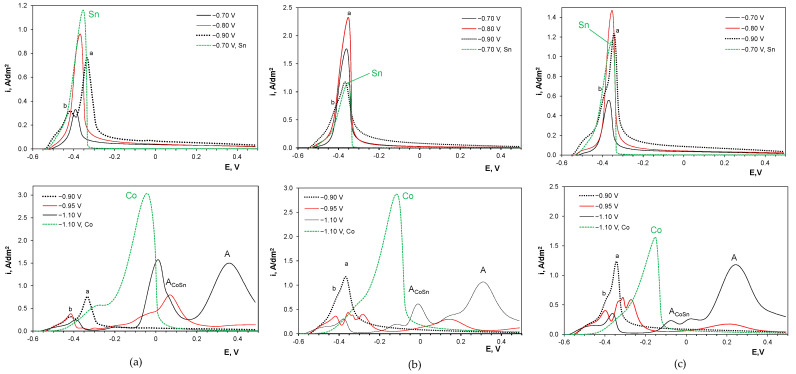
ASLV of deposits accumulated for 40 s on glassy carbon in gluconate baths: (**a**) chloride, (**b**) chloride–sulfate, (**c**) sulfate (green dashed lines correspond to dissolution of single metals as references). Anode peak labels are explained in the text. E vs. Ag/AgCl.

The observed phenomenon of delayed nucleation can be attributed to the formation of the CoSn phase during electrodeposition. Anodic sweep voltammetric curves shown in Figure 8 confirm this, since their course changes drastically for the accumulation potential of −0.95 V. At more positive deposition potentials, only tin oxidation peaks at about −0.4 V were observed due to no or very low cobalt incorporation. The development of two Sn peaks for the accumulation potential of −0.9 V demonstrates early variations in the composition of the deposits, which is more evident in the chloride bath. The double tin anodic peaks can be attributed to the dissolution of tin from the pure metal phase (a) and the CoSn phase (b). The latter process requires a higher overpotential to remove tin ions from the crystal lattice of the intermetallic phase. At the transition deposition potential of −0.95 V, tin seems to dissolve from different substrates, suggested by the existence of a wide triple anodic peak (b). These oxidation processes are more pronounced in the sulfate-containing baths, indicating differences in the electrochemistry of sulfate and chloride systems. As the deposition potential becomes more negative, the intensities of the tin anodic peaks decrease, while new broad anodic peaks (A_CoSn_) appear at more positive potentials (of about 0 V). These correspond to the dissolution of cobalt from the CoSn phase, similar to observations in the CV anodic branches (Figure 2). This is in good accordance with the previous data reporting the dissolution of cobalt-rich phases from the deposits at the potential range of −0.2–0 V under agitation conditions [4]. One more anodic peak (A) with a maximum at 0.3–0.4 V was developed. It was not detected during cyclic voltammetric measurements (Figure 2). This peak can be attributed to the secondary reaction of cobalt oxidation (to oxide), which occurs in this potential range in alkaline gluconate electrolytes [56]. The anodic peak A was more pronounced at more negative potentials, where the more intense evolution of hydrogen can rapidly increase the pH of the non-agitated electrolyte at the electrode surface. This concurrent process of hydrogen evolution can be additionally catalyzed by the CoSn phase [4] produced under such conditions. 

### 2.6. Surface Wettability

**Figure 9:**
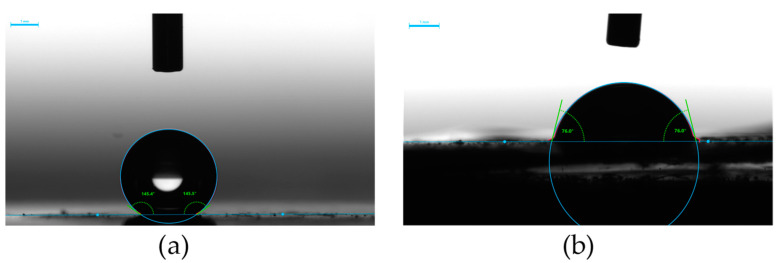
Exemplary views of liquid droplets on the deposit surface: (**a**) water, (**b**) diiodomethane. Deposition conditions: sulfate–gluconate bath, −0.8 V. Scale bar 1 mm.

**Figure 10:**
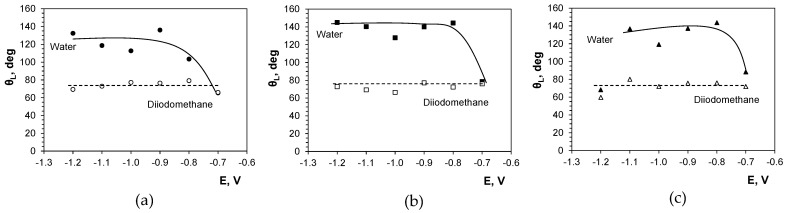
Influence of deposition potential on the wettability of the deposits produced in gluconate baths: (**a**) chloride, (**b**) chloride–sulfate, (**c**) sulfate. E vs. Ag/AgCl.

The wettability of solids is predominantly governed by two factors, i.e., surface topography and chemical composition. A combination of a micro- and nanoscale superficial structure and/or a nonpolar surface chemistry helps trap large amounts of air, reducing the attractive forces between the solid surface and liquid droplets. This prevents the liquid from spreading over the surface and penetrating into its irregularities [57]. Figure 9 and Figure 10 show water and diiodomethane contact angles on the electrodeposits produced in this study. They indicate no significant effect of the deposition potential and alloy composition on the deposit wettability by nonpolar liquid (diiodomethane), as the contact angles are at similar levels of 73 ± 5°, demonstrating relatively stable oleophilic properties. Some effect can be seen for the polar liquid (water). The tin layers produced at −0.7 V, with relatively low roughness, can be classified as weakly hydrophilic, since the contact angles are 65°, 78°, and 88° for chloride, chloride–sulfate, and sulfate baths, respectively. This shows a decreasing wetting tendency for the deposits produced from sulfate-containing baths. Incorporation of cobalt at more negative potentials results in changed deposit composition and increased deposit roughness, thus increasing the water contact angles to 103–144°, making the surface evidently hydrophobic.

**Figure 11:**
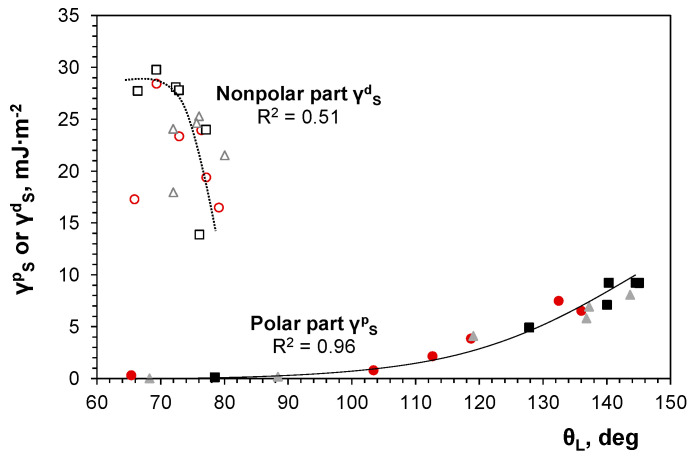
Dependence of polar and nonpolar parts of free surface energies of deposits produced from gluconate baths: chloride—circles; chloride–sulfate—squares; sulfate—triangles.

The partial (Figure 11) and total (Table 4) surface free energies of the alloy coatings were calculated. The surface free energy of the alloy deposits was determined using the Owens–Wendt–Rabel–Kealble method [57]:

(9) $$2(\sqrt{\gamma_{S}^{d}\gamma_{L}^{d}}+\sqrt{\gamma_{S}^{p}\gamma_{L}^{p}})=\gamma_{L}\left(1+cos\theta_{L}\right)$$

where the total surface free energy *γ* is assumed to be the sum of its polar *γ^p^* and nonpolar (dispersion) *γ^d^* components:

γ = *γ^p^* + *γ^d^* (10)

Subscripts *S* and *L* are related to solid–gas and liquid–gas interfaces, respectively.

The total surface free energy of the deposit was calculated based on the contact angles for demineralized water *θ_W_* and diiodomethane *θ_D_* as reference liquids using the following equation for the nonpolar part [58]:

(11) $$\sqrt{\gamma_{S}^{d}}=\frac{\gamma_{D}\left(cos\theta_{D}+1\right)-\sqrt{\frac{\gamma_{D}^{p}}{\gamma_{W}^{p}}}{\;\gamma }_{W}(cos\theta_{W}+1)}{2\left(\sqrt{\gamma_{D}^{d}}-\sqrt{\frac{\gamma_{D}^{p}\gamma_{W}^{d}}{\gamma_{W}^{p}}}\right)}$$

and rearranged Equation (9) for the polar part:

(12) $$\sqrt{\gamma_{S}^{p}}=\frac{\gamma_{W}\left(cos\theta_{W}+1\right)-2\sqrt{\gamma_{P}^{d}\gamma_{W}^{d}}}{2\sqrt{\gamma_{W}^{P}}}$$

where *γ_W_ =* 72.8 mJ/m^2^, *γ^p^_W_* = 51.0 mJ/m^2^, *γ^d^_W_* = 21.8 mJ/m^2^ for water and *γ_D_ =* 50.8 mJ/m^2^, *γ^p^_D_* = 2.3 mJ/m^2^, *γ^d^_D_* = 48.5 mJ/m^2^ for diiodomethane.

The values of the nonpolar parts were higher than those of the polar parts, while the total surface energies were quite similar (32 ± 7 mJ/m^2^), showing differences in the liquid-deposit behavior. The contributions of the individual components to the total surface energy are primarily dependent on the intermolecular forces between the liquid and solid contacting phases. Nonpolar forces have a universal character and occur in all substances; thus, they always contribute to the total surface free energy. Polar forces, on the other hand, exist only in specific substances composed of particles that exhibit electrostatic interactions, such as those with metallic, ionic, or covalent polarized bonds. Consequently, the surface energy depends on the chemical composition of the phases, in accordance with the Young wettability model for flat solid surfaces [57].

The surface free energy basically represents the scale of unrealized binding energy and, therefore, should be independent of the surface roughness. However, since the surface energy is calculated using experimentally determined contact angles, surface irregularities in a micro- and/or nanometric scale do affect the wetting behavior of the surface by liquids. This relationship is predicted by the Wenzel model, in which liquid droplets penetrate into the rough surface structure and fill the valleys [59]:

cos*θ_W_* = *r*·cos*θ_Y_* (13)

Therefore, the roughness *r* (*r* > 1) modifies the surface wettability *θ_W_*, depending on the initial property of the flat substrate material *θ_Y_*.

Liquid droplets can also remain suspended on the tops of a rough surface, preventing them from entering the irregularities of the solid substrate due to the existence of air cushions trapped in the surface pores. In such a case, the contact angle between the droplet and the composite solid interface can be described by the Cassie–Baxter model [60]:

cos*θ_CB_* = *f*_1_·cos*θ_Y_*_1_
*+ f*_2_·cos*θ_Y_*_2_
*= f*_1_·(cos*θ_Y_*_1_ +1) − 1 (14)

where *f*_1_ and *f*_2_ are surface fractions of a two-component (1—solid; 2—air) composite rough surface, respectively, whereas *θ_Y_*_1_ and *θ_Y_*_2_ are particular Young contact angles at liquid–solid and liquid–air interfaces (*θ_Y_*_2_ = 180°).

The intermediate state of heterogeneous wetting, where the liquid droplet partially wets the surface and partially sits on air pockets, was discussed by Marmur [61]. For such a case, the following relationship was proposed:

cos*θ_M_* = *r_f_*·*f_s_*·cos*θ* + *f_s_* − 1 (15)

where *r_f_* is the roughness of the solid that touches the liquid, *f_s_* is the area fraction of the solid phase of the rough surface, and *θ* is the intrinsic contact angle between the droplet and the solid phase.

An analysis of the results obtained in this study, in terms of the aforementioned wettability models, leads to the conclusion that the parts of the surface free energy are influenced by the competitive effects of surface chemistry and rough topography according to the Cassie–Baxter or Marmur approaches. Water droplets with higher surface tension may partially be held on the tops of surface irregularities. The wettability of the alloy deposits by nonpolar liquid seems to follow the Wenzel model since the high *γ^d^* part and the lower surface tension of diiodomethane enable its penetration into surface irregularities, favored by nonpolar liquid–solid interactions. These statements are supported by the corresponding scattering of *γ^d^* and *γ^p^* values on the contact angles for all coatings. Thus, it is acceptable to conclude that the wettability of the coatings results more from the surface roughness than from their chemical composition.

### 2.7. Nucleation and Growth of Deposits

The initial stages of the deposition of the metallic phase were analyzed using the chronoamperometric curves shown in Figure 7. Instantaneous and progressive nucleation of 3D hemispherical nuclei are most often considered according to the formalisms proposed by Sharifker and Hills [62]. They classify the nucleation type by comparing experimental results with theoretical predictions. According to this approach, instantaneous nucleation (IN) involves the simultaneous generation of nuclei followed by their growth at the same rate. For this case, the following diagnostic equation was established:

(16) $${\left(\frac{i}{i_{max}}\right)}^{2}=\frac{1.9542}{t/t_{max}}{\left[1-exp\left(-1.2564\frac{t}{t_{max}}\right)\right]}^{2}$$

In turn, progressive nucleation (PN) involves the continuous formation of nuclei, with their growth to different sizes and/or at different rates. This scenario is described by the following diagnostic equation:

(17) $${\left(\frac{i}{i_{max}}\right)}^{2}=\frac{1.2254}{t/t_{max}}{\left[1-exp\left(-2.3367{\left[\frac{t}{t_{max}}\right]}^{2}\right)\right]}^{2}$$

**Figure 12:**
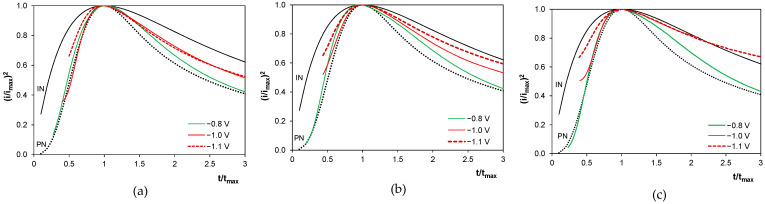
Theoretical and experimental curves for instantaneous (IN) and progressive (PN) nucleation models of metals deposited from gluconate baths: (**a**) chloride, (**b**) chloride–sulfate, (**c**) sulfate. E vs. Ag/AgCl.

Figure 12 shows exemplary plots for both nucleation models alongside the experimental chronoamperometric data for deposition potentials below and above −0.9 V, representing primarily tin deposition and tin with CoSn phase codeposition, respectively. The data for −0.9 V are disregarded due to a weakly marked maximum I in both sulfate-containing baths (Figure 7b,c). For all baths, the formation of the metal phase followed the progressive nucleation model for tin at −0.8 V. However, the codeposition of metals complicates the nucleation stage, causing deviations from the progressive nucleation reference curve at *t/t_max_* values greater than 1. These shifts in the experimental curves are more pronounced in the presence of sulfate ions, indicating a possible transition towards instantaneous nucleation. This behavior aligns well with the data for pure tin (PN) [26] and pure cobalt nucleation (PN or IN) [42,63] in gluconate baths. It should be also noted that the Sharifker and Hills model is primarily valid for fast electrochemical processes with high exchange current densities [62], such as tin deposition, but not necessarily for cobalt deposition. 

**Figure 13:**
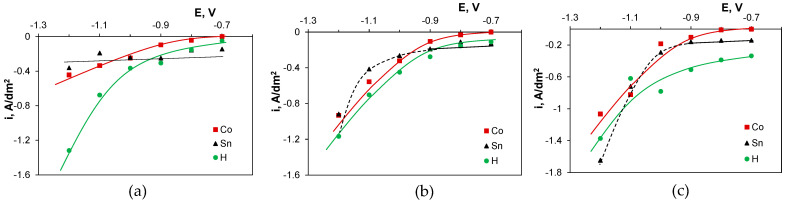
Partial polarization curves calculated based on potentiostatic deposition data from gluconate baths: (**a**) chloride, (**b**) chloride–sulfate, (**c**) sulfate. E vs. Ag/AgCl.

The nucleation stage is followed by the growth of the metallic phase. Figure 13 shows partial polarization curves calculated from the potentiostatic deposition data (Figure 3). They clearly show the reduction of tin(II) species under limiting current in the chloride bath at all deposition potentials, while in the presence of sulfate ions, this behavior occurs only at more positive potentials, where cobalt incorporation is hindered. This phenomenon can be correlated with the transition of the nucleation type from progressive to instantaneous in the presence of sulfate anions at longer deposition times, i.e., during the nucleation of metallic layers successively on the previously formed metal grains. 

Competition between nucleation and growth of nuclei determines the morphology and structure of metallic coatings [44,55]. Pure tin is known to easily form dendrites during electrodeposition [25,26,27,32,52,53]. This process can be inhibited by the incorporation of cobalt, which is somewhat less efficient in the sulfate-containing baths (Figure 3b). It enhances more rapid tin nucleation and growth (Figure 3a) with a texture different from that observed for the phase produced in the chloride electrolyte (Table 3).

## 3. Materials and Methods

Codeposition of the alloys was carried out from three gluconate solutions: chloride, chloride–sulfate, and sulfate with detailed compositions shown in Table 5. The pH of all baths was 3.5. All reagents (from Avantor Performance Materials Poland S.A., Gliwice, Poland) used were of analytical-grade purity.

The ionic speciation for each bath composition was calculated using the equilibria data shown in Table 1 with the free HySS2009 software. The principle of calculating the equilibrium ionic composition of solutions as a function of pH involves developing polynomial equations for the mass balance of specific species. This is done using chemical equilibrium equations, dissociation constants, and complex stability constants to determine the concentrations of all ions present at a given pH. Details of the HySS program’s operation can be found in [64].

Electrochemical potentiodynamic measurements were carried out in a three-electrode cell using a glassy carbon working electrode (0.2 cm^2^), a platinum plate (2 cm^2^) as the counter electrode, and an Ag/AgCl electrode as the reference electrode (all potentials in the text are referred to this electrode). Cyclic voltammetry (CV) with a sweep rate of 10 mV/s was initiated from a potential of 0.5 V vs. Ag/AgCl and continued towards more negative values during the first scan. The backward scan was finished at the same potential as the initial one. Cathodic polarization curves were recorded in the same system using blank solutions containing potassium salts instead of tin and cobalt salts. Chronoamperometric measurements were performed for 40 s at various potentials. Anodic stripping voltammetry was further conducted at a scan rate of 10 mV/s without removing the sample from the solution. The Co-Sn alloys were deposited at constant potentials for 60 min in the same system, using copper plates (0.44 cm^2^) as cathode substrates. Before each experiment, the glassy carbon electrode with a mirror finish was chemically cleaned, while the copper sheets were chemically polished in a mixture of concentrated acids (HNO_3_/H_3_PO_4_/CH_3_COOH with a volume ratio of 1:3:1). All electrochemical experiments were performed using an Autolab potentiostat/galvanostat (PGSTAT302N, Metrohm; Herisau, Switzerland) and non-agitated solutions with a volume of 20 cm^3^. 

The cathodic current efficiency *η* of the alloy deposition was calculated based on the mass of the deposit *m* (Figure 3a), its chemical composition in weight percentages *P_Co_, P_Sn_* (Figure 3b), and the total electrical charge *Q* that flowed through the circuit during electrolysis. The following formulas were used for this calculation:

(18) $$\eta =\frac{m}{m_{t}}\cdot 100\%$$

and

(19) $$m_{t}=\frac{Q\cdot 100\%}{2F\left(\frac{P_{Co}}{M_{Co}}+\frac{P_{Sn}}{M_{Sn}}\right)}$$

where *m_t_* is the theoretical mass of the deposits, *F* is the Faraday constant, and *M_Co_* and *M_Sn_* are the molar masses of the individual metals.

The morphology of the coatings was examined using a scanning electron microscope (SEM) (Hitachi SU-70; Tokyo, Japan). The elemental composition of the alloys was determined using energy-dispersive X-ray spectroscopy *(*EDS) (Thermo Scientific EDS system; Waltham, MA, USA). The analysis was conducted on at least three areas of about 8500 μm^2^ each (at the same magnifications) on the central part of the deposit surface to avoid disturbances in the layer composition caused by edge effects during electrodeposition. Phase composition was analyzed by X-ray diffractometry (XRD) (Rikagu diffractometer, CuK_α_ radiation; Tokyo, Japan). The wettability of the deposits was examined using water and diiodomethane as polar and nonpolar liquids, respectively. Contact angles *θ_L_* of stationary droplets were measured with a goniometer (DSA25, Krűss; Hamburg, Germany). A scaler installed at a liquid container injector was used to control the volume of the droplet. The contact angle tester featured a needle that could hold very small droplets (1.8 ± 0.1 μL), which were dropped on the sample under gravity. This was followed by taking a series of snapshots and automatic measurements of the contact angles until stable values were obtained. 

All experiments were performed at ambient temperature (19 ± 1 °C). 

## 4. Conclusions

The codeposition of alloys from gluconate baths containing cobalt(II) and tin(II) was investigated. Two-phase alloys with tetragonal tin and the uncommon tetragonal CoSn phase were obtained. This was confirmed by diffraction analysis and electrochemical investigations. The mass of deposits, the cathodic current efficiencies, and the morphology and texture of the layers depended on the deposition potential and the anions (chloride, sulfate) in the electrolyte. This resulted in porous deposits with highly developed surfaces featuring different hierarchical topologies and relatively similar cobalt contents. Consequently, the alloys exhibited dual wettability, showing hydrophobicity and stable oleophilicity. The electrodeposition of practically pure tin at the most positive potentials used proceeded via progressive nucleation, resulting in the growth of the preferred orientation (321) plane. In contrast, the nucleation and growth of the tin phase codeposited with the CoSn phase were found to depend on the type of anion present in the electrolyte. Sulfate ions favored a transition from a progressive to an instantaneous mechanism of 3D nuclei formation and the growth of the tin (220) preferential plane. Conversely, progressive nucleation appeared responsible for the preferential growth of the (200) and (101) planes in the presence of chloride anions. The texture of the CoSn phase was independent of the bath speciation.

The obtained results also indicate two directions for further studies and potential applications of the Co-Sn alloys produced from gluconate baths. The first is the possibility of producing slippery lubricant-infused coatings. This is supported by the porous surface structure of the coatings, which features a highly developed surface of columnar or dendritic structures with oleophilic properties. This should facilitate the infiltration and retention of low-surface-tension liquid lubricants within the surface pores. The hydrophobic properties, in turn, will prevent water from penetrating the cavities. Such properties are essential for achieving high mobility and very low wetting angle hysteresis of liquid droplets (immiscible with the lubricant encapsulated in surface protrusions) on surfaces exhibiting self-cleaning, anti-corrosion, and anti-fouling properties. However, this behavior is only sustained as long as the lubricant remains infused into the surface pores. Ensuring this retention is the main challenge for truly sustainable applications of such slippery surfaces. Therefore, understanding the mechanisms for improving the ability of these surfaces to retain lubricant (whether through mechanical trapping in textures, impregnation with emerging features, and/or due to intermolecular interactions) is fundamental for optimizing SLIPS coating production processes.

On the other hand, porous oleophilic Co-Sn alloy structures have potential applications as electrode materials for Li-ion batteries. This aspect is particularly intriguing due to the lack of electrochemical studies on the capacity, cyclic performance, and stability of the unusual tetragonal CoSn phase as an anode material. These properties are influenced by the material’s electrochemical characteristics, including its ability to intercalate lithium ions, conductivity, and mechanical integrity during cycling. Understanding the correlations between the alloy composition (involving this uncommon phase), microstructure, and electrochemical behavior is crucial for optimizing anode performance.

The above considerations highlight the important factors necessary to recognize the specific requirements of Co-Sn alloys as new materials for innovative applications. Accordingly, this work provides systematic basic information on the composition and microstructure of coatings produced from various gluconate electrolytes. However, further research is needed to verify and develop their properties for specific purposes.

## Figures and Tables

**Table 1:** Equilibrium constants at 298 K [33,34,35,36,37].

Reaction	Equilibrium Constant (Logarithmic Values)
Co^2+^ + H_2_O ↔ CoOH^+^ + H^+^	−9.687
Co^2+^ + 2H_2_O ↔ Co(OH)_2_ + 2H^+^	−18.794
Co^2+^ + 3H_2_O ↔ Co(OH)_3_^−^ + 3H^+^	−31.491
Co^2+^ + 4H_2_O ↔ Co(OH)_4_^2−^ + 4H^+^	−46.288
2Co^2+^ + H_2_O ↔ Co_2_OH^3+^ + H^+^	−10.997
Co^2+^ + SO4^2−^ ↔ CoSO_4_	2.36
Co^2+^ + Cl^−^ ↔ CoCl^+^	0.22
Co^2+^ + 2Cl^−^ ↔ CoCl_2_	−3.95
Co^2+^ + 3Cl^−^ ↔ CoCl_3_^−^	−3.02
Co^2+^ + 4Cl^−^ ↔ CoCl_4_^2−^	−9.06
Co^2+^ + Glu^−^ ↔ CoGlu+	2.31
Co^2+^ + Glu^−^ ↔ CoH_−1_Glu + H^+^	−4.96
Co^2+^ + Glu^−^ ↔ CoH_−2_Glu^−^ + 2H^+^	−13.29
Co^2+^ + 3Glu^−^ ↔ CoH_−1_Glu_3_^2−^ + H^+^	−1.27
Co^2+^ + 3Glu^−^ ↔ CoH_−2_Glu_3_^3−^ + 2H^+^	−9.21
2Co^2+^ + 2Glu^−^ ↔ Co_2_H_−3_Glu_2_^−^ + 3H^+^	−17.89
Co^2+^ + NH_3_ ↔ Co(NH_3_)^2+^	1.99
Co^2+^ + 2NH_3_ ↔ Co(NH_3_)_2_^2+^	3.50
Co^2+^ + 3NH_3_ ↔ Co(NH_3_)_3_^2+^	4.43
Co^2+^ + 4NH_3_ ↔ Co(NH_3_)_4_^2+^	5.07
Co^2+^ + 5NH_3_ ↔ Co(NH_3_)_5_^2+^	5.13
Co^2+^ + 6NH_3_ ↔ Co(NH_3_)_6_^2+^	4.39
Sn^2+^ + H_2_O ↔ SnOH^+^ + H^+^	−4.1
Sn^2+^ + 2H_2_O ↔ Sn(OH)_2_ + 2H^+^	−7.8
Sn^2+^ + 3H_2_O ↔ Sn(OH)_3_^−^ + 3H^+^	−17.6
Sn^2+^ + SO_4_^2−^ ↔ SnSO_4_	1.29
Sn^2+^ + 2SO_4_^2−^ ↔ Sn(SO_4_)_2_^2−^	1.65
Sn^2+^ + Cl^−^ ↔ SnCl^+^	1.42
Sn^2+^ + 2Cl^−^ ↔ SnCl_2_	2.18
Sn^2+^ + 3Cl^−^ ↔ SnCl_3_^−^	2.33
Sn^2+^ + 4Cl^−^ ↔ SnCl_4_^2−^	2.03
Sn^2+^ + Glu^−^ ↔ SnGlu^+^	3.01
Sn^2+^ + 2Glu^−^ ↔ SnGlu_2_	5.29
H+ + SO_4_^2−^↔ HSO_4_^−^	1.12
H+ + Cl^−^ ↔ HCl	−7.0
H+ + Glu^−^ ↔ HGlu	3.35
H+ + NH_3_ ↔ NH_4_^+^	9.22

**Table 2:** Thermodynamic data for Co_x_Sn_y_ phases [47] and corresponding changes in quasi-rest potentials of cobalt and tin electrodes (298 K).

Phase	Δ*G^o^*, kJ·mol^−1^	Shift of Quasi-Rest Potential, V		Difference in Quasi-Rest Potentials, V
		$∆E_{o,Co}=-\frac{∆G^{o}}{2xF}$	$∆E_{o,Sn}=-\frac{∆G^{o}}{2yF}$	
CoSn	−19.0	0.099	0.099	0.130
CoSn_2_	−12.7	0.066	0.033	0.097
α-Co_3_Sn_2_	−16.8	0.029	0.044	0.145
β-Co_3_Sn_2_	−16.5	0.029	0.043	0.144

**Table 3:** Texture coefficients calculated based on the XRD data and standard used (tin JCPDS Card No. 00-004-0673, tetragonal CoSn phase [4]). E vs. Ag/AgCl.

Deposition Potential, V	Texture Coefficient T_c_(*hkl*)						
	Sn(200) 30.7°	Sn(101) 32.0°	Sn(220) 43.9°	Sn(321) 64.7°	CoSn (100) 28.9°	CoSn(101) 32.8°	CoSn(110) 41.3°
Chloride bath							
−0.7	0.1	0.1	0.9	2.9	nd	nd	nd
−0.9	1.3	1.3	1.0	0.4	0.9	1.5	0.7
−1.1	2.3	1.7	0.0	0.0	0.5	1.5	1.0
−1.2	2.2	1.8	0.0	0.0	0.7	1.7	0.6
Chloride–sulfate bath							
−0.7	0.4	0.3	0.8	2.5	nd	nd	nd
−0.9	1.4	0.8	1.5	0.3	0.8	1.5	0.7
−1.1	1.2	0.8	1.6	0.4	0.9	1.4	0.6
−1.2	0.9	0.6	2.0	0.5	0.7	1.5	0.7
Sulfate bath							
−0.7	0.4	0.3	0.8	2.5	nd	nd	nd
−0.9	0.8	1.0	1.6	0.6	0.8	1.6	0.6
−1.1	0.8	0.8	1.7	0.7	0.8	1.4	0.8
−1.2	0.8	0.8	2.0	0.4	0.8	1.3	0.9

nd—not detectable.

**Table 4:** Total free surface energies of Co-Sn deposits produced from gluconate baths.

Deposition Potential, V (Ag/AgCl)	Total Free Surface Energy *γ_S_*, mJ∙m^−2^		
	Chloride	Chloride–Sulfate	Sulfate
−0.8	25.8	37.3	33.3
−0.9	30.4	31.1	31.5
−1.0	31.2	32.6	28.2
−1.1	28.4	39.0	27.3
−1.2	35.9	37.0	26.4

**Table 5:** Composition of gluconate baths.

Component	Bath Composition, M		
	Chloride	Chloride–Sulfate	Sulfate
SnCl_2_	0.05	0.05	-
SnSO_4_	-	-	0.05
CoCl_2_	0.1	-	-
CoSO_4_	-	0.1	0.1
NH_4_Cl	0.5	-	-
(NH_4_)_2_SO_4_	-	0.5	0.5
C_6_H_11_O_7_Na	0.2	0.2	0.2
H_3_BO_3_	0.5	0.5	0.5

## Data Availability

Data are contained within the article or available on personal request.

## References

[1] Brenner A. (1963). Electrodeposition of Alloys.

[2] Abd El Rehim S.S., Refaey S.A., Schwitzgebelf G., Taha F., Saleh M.B. (1996). Electrodeposition of Sn-Co alloys from gluconate baths. J. Appl. Electrochem..

[3] Sujatha M., Sabitha R., Pushpavanam M. (2000). Electrodeposited cobalt-tin alloy from a neutral gluconate bath. Trans. IMF.

[4] Gomez E., Guaus E., Torrent J., Alcobe X., Valles E. (2001). Cobalt-tin electrodeposition from sulfate-gluconate baths. J. Appl. Electrochem..

[5] Vinokurov E.G. (2010). Prognostication of the composition of a solution for electrodeposition of Sn–Co alloy and determination of its color characteristics. Russ. J. Appl. Chem..

[6] Medvedev G.I., Makrushin N.A. (2012). Electrodeposition of tin–cobalt alloy from a sulfate electrolyte with organic additives. Russ. J. Appl. Chem..

[7] Valkova T., Krastev I. (2016). Influence of glycine on the electrochemical deposition of Sn-Co alloy from gluconate electrolyte. Bulg. Chem. Comm..

[8] Refaey S.A.M. (1999). Corrosion of Sn–Co alloy in alkaline media and the effect of Cl^−^ and Br^−^ ions. Appl. Surf. Sci..

[9] Zhang J.L., Gu C.D., Fashu S., Tong Y.Y., Huang M.L., Wang X.L., Tua J.P. (2015). Enhanced corrosion resistance of Co-Sn alloy coating with a self-organized layered structure electrodeposited from deep eutectic solvent. J. Electrochem. Soc..

[10] Pereira N.M., Sousa C.T., Pereira C.M., Araujo J.P., Silva A.F. (2017). Enhanced properties of Co-Sn coatings electrodeposited from choline chloride-based deep eutectic solvents. Cryst. Growth Des..

[11] Cho S.K., Han H.S., Lee C.K., Ahn C.I., Park J.I. (2003). Cobalt-tin plating in a pyrophosphate bath. Mat. Sci. Forum..

[12] Georgiou E.P., Buijnsters J.G., Wang H., Drees D., Basak A.K., Celis J.P. (2015). Nanostructured gradient Co-Sn electrodeposits as alternative to Sn connector contacts. Surf. Coat. Technol..

[13] Vitina I., Belmane V., Krumina A., Rubene V. (2011). Changes in the phase composition and structure of electrodeposited Sn-Co alloys in Sn-Co/Sn layer systems upon heating. Surf. Coat. Technol..

[14] Hy R.Z., Liu H., Zeng M.Q., Liu J.W., Zhu M. (2012). Progress on Sn-based thin film anode materials for lithium-ion batteries. Chin. Sci. Bull..

[15] Fan X.-Y., Yang F.-Z., Sun S.-G. (2007). Fabrication and properties of macroporous tin–cobalt alloy film electrodes for lithium-ion batteries. J. Power Sources.

[16] Fan X.Y., Ke F.S., Wei G.Z., Huang L., Sun S.G. (2009). Sn-Co alloy anode using porous Cu as current collector for lithium ion battery. J. Alloys Compd..

[17] Ui K., Kikuchi S., Jimba Y., Kumagai N. (2011). Preparation of Co–Sn alloy film as negative electrode for lithium secondary batteries by pulse electrodeposition method. J. Power Sources.

[18] Gnanamuthu R.M., Jo Y.N., Lee C.W. (2013). Brush electroplated CoSn_2_ alloy film for application in lithium-ion batteries. J. Alloys Compd..

[19] Groult H., El Ghallali H., Barhoun A., Briot E., Perrigaud L., Hernandorena S., Lantelme F. (2010). Preparation of Co–Sn alloys by electroreduction of Co(II) and Sn(II) in molten LiCl–KCl. Electrochim. Acta.

[20] Liu Y., Lu H., Kou X. (2019). Electrodeposited Ni-Co-Sn alloy as a highly efficient electrocatalyst for water splitting. Int. J. Hydrogen Energy.

[21] Chandran A.M., Karumuthil S.C., Singh A.K., Prasad B.L.V. (2024). Electrodeposited Co-Mn-Sn multicomponent alloy as an efficient electrocatalyst for hydrogen evolution reaction. Int. J. Hydrogen Energy.

[22] Survila A., Mockus Z., Kanapeckaite S., Stalnionis G. (2012). Kinetics of Sn(II) reduction in acid sulphate solutions containing gluconic acid. J. Electroanal. Chem..

[23] Mockus Z., Norkus E., Vaitkus R., Kalinauskas P., Grinciene G., Tamasauskaite-Tamasiunaite L. (2020). Inhibition of Sn(II) oxidation by air oxygen in acidic gluconate-containing solutions. J. Electrochem. Soc..

[24] Guaus E., Torrent-Burgues J. (2003). Tin–zinc electrodeposition from sulphate–gluconate baths. J. Electroanal. Chem..

[25] Rudnik E., Chowaniec G. (2018). Effect of organic additives on electrodeposition of tin from acid sulfate solution. Metall. Foundry Eng..

[26] Rudnik E. (2013). Effect of anions on the electrodeposition of tin from acidic gluconate baths. Ionics.

[27] Rudnik E., Włoch G. (2013). Studies on the electrodeposition of tin from acidic chloride-gluconate solutions. Appl. Surf. Sci..

[28] Rudnik E. (2014). The influence of sulfate ions on the electrodeposition of Ni-Sn alloys from acidic chloride-gluconate baths. J. Electroanal. Chem..

[29] Kuzmann E., Felner I., Sziraki L., Stichleutner S., Homonnay Z., El-Sharif M., Chisholm C.U. (2022). Magnetic anisotropy and microstructure in electrodeposited quaternary Sn-Fe-Ni-Co alloys with amorphous character. Materials.

[30] Tripathi D., Ray P., Singh A.V., Kishore V., Singh S.L. (2023). Durability of slippery liquid-infused surfaces: Challenges and advances. Coatings.

[31] Kaden N., Schlimbach R., Garcia A.R., Droder K. (2023). A systematic literature analysis on electrolyte filling and wetting in lithium-ion battery production. Batteries.

[32] Rudnik E., Dashbold N. (2015). Electrolytic recovery of tin from solder alloy in acid chloride solutions. Rudy Met. Rec..

[33] The IUPAC Stability Constants Database; Academic Software and IUMAC, 1992–2000. https://www.acadsoft.co.uk.

[34] Ashton F., Pickering W.F. (1970). Cobalt(II) gluconate complexes. Aust. J. Chem..

[35] Pettine M., Millero F.J., Macchi G. (1981). Hydrolysis of tin(II) in aqueous solutions. Anal. Chem..

[36] Müller B., Seward T.M. (2001). Spectrophotometric determination of the stability of tin (II) chloride complexes in aqueous solution up to 300 °C. Geochim. Cosmochim. Acta.

[37] Kochkodan V., Darwish N.B., Hilal N., Kabay N., Bryjak M., Hilal N. (2015). The chemistry of boron in water. Boron Separation Processes.

[38] Smith P.J. (1998). Chemistry of Tin.

[39] Soto A.B., Arce E.M., Palomar-Pardavé M., Gonzalez I. (1996). Electrochemical nucleation of cobalt on glassy carbon electrode from ammonium chloride solutions. Electrochim. Acta.

[40] Grujicic D., Pesic B. (2004). Electrochemical and AFM study of cobalt nucleation mechanisms on glassy carbon from ammonium sulfate solutions. Electrochim. Acta.

[41] Łętowski F., Niemiec J. (1969). Diagrams of electrochemical equilibria E-pH at 25 °C. Part IV. Co-H_2_O-NH_3_-H_2_SO_4_ system. Rocz. Chem..

[42] Rudnik E., Dashbold N. (2019). Effect of Cl^−^ and SO_4_^2−^ ions on electrodeposition of cobalt from acidic gluconate solutions. Russ. J. Electrochem..

[43] Tripkovic D.V., Strmcnik D., van der Vliet D., Stamenkovic V., Markovic N.M. (2008). The role of anions in surface electrochemistry. Farad. Discuss..

[44] Laszlo P. (2021). Electrochemical Methods of Nanostructure Preparation.

[45] Matsushima J.T., Trivinho-Strixino F., Pereira E.C. (2006). Investigation of cobalt deposition using the electrochemical quartz crystal microbalance. Electrochim. Acta.

[46] Kröger F.A. (1978). Cathodic deposition and characterization of metallic or semiconducting binary alloys or compounds. J. Electrochem. Soc..

[47] Liu L., Andersson C., Liu J. (2004). Thermodynamic assessment of the Sn-Co lead free solder system. J. Electr. Mater..

[48] Ishida K., Nishizawa T. (1991). The Co-Sn (cobalt-tin) system. J. Phase Equilib..

[49] Tamura N., Kato Y., Mikami A., Kamino M., Matsuta S., Fujitani S. (2006). Study on Sn-Co alloy anodes for lithium secondary batteries. I. Amorphous system. J. Electrochem. Soc..

[50] Gul H., Uysal M., Cetinkaya T., Guler M.O., Alp A., Akbut H. (2014). Preparation of Sn-Co alloy electrode for lithium ion batteries by pulse electrodeposition. Int. J. Hydrogen Energy.

[51] Jaen J., Varsanyi M.L., Czako-Nagy I., Buzas A., Vertes A., Kiss L. (1984). Structural studies of electrodeposited cobalt-tin alloys. Electrochim. Acta.

[52] Eckold P., Niewa R., Hügel W. (2014). Texture of electrodeposited tin layers and its influence on their corrosion behavior. Microel. Reliab..

[53] Eckold P., Sellers M.S., Niewa R., Hügel W. (2015). The surface energies of β-Sn—A new concept for corrosion and whisker mitigation. Microel. Reliab..

[54] Garret C.S., Massalski T.B. (1980). Structure of Metals.

[55] Nasirpouri F. (2017). Electrodeposition of Nanostructured Materials.

[56] Casella I.G., Contursi M. (2012). Cobalt oxide electrodeposition on various electrode substrates from alkaline medium containing Co–gluconate complexes: A comparative voltammetric study. J. Solid State Electrochem..

[57] Honciuc A. (2021). Chemistry of Functional Materials, Surfaces and Interfaces.

[58] Jańczuk B., Białopiotrowicz T. (1989). Surface free-energy components of liquids and low energy solids and contact angles. J. Coll. Interf. Sci..

[59] Wenzel R.N. (1936). Resistance of solid surfaces to wetting by water. Ind. Eng. Chem..

[60] Cassie A.B.D., Baxter S. (1944). Wettability of porous surfaces. Trans. Farad. Soc..

[61] Marmur A. (2003). Wetting on hydrophobic rough surfaces: To be heterogeneous or not to be?. Langmuir.

[62] Sharifker B.R., Hills G. (1983). Theoretical and experimental studies of multiple nucleation. Electrochim. Acta.

[63] Rudnik E. (2024). Effect of pH-dependent bath speciation on cobalt electrodeposition from sulfate-gluconate solutions. Trans. Nonferr. Met. Soc. China.

[64] Alderighi L., Gans P., Ienco A., Peters D., Sabatini A., Vacca A. (1999). Hyperquad simulation and speciation (HySS): A utility program for the investigation of equilibria involving soluble and partially soluble species. Coord. Chem. Rev..

